# Public support for new tobacco control measures in Poland: A cross-sectional survey 2024

**DOI:** 10.18332/tpc/196135

**Published:** 2024-12-27

**Authors:** Justyna Grudziąż-Sękowska, Kuba Bartłomiej Sękowski, Zuzanna Grześczyk-Nojszewska, Radosław Sierpiński, Janusz Ostrowski, Jarosław Pinkas, Mateusz Jankowski

**Affiliations:** 1 Public Health School, Centre of Postgraduate Medical Education, Warsaw, Poland; 2 Faculty of Medicine, Collegium Medicum, Cardinal Stefan Wyszyński University, Warsaw, Poland

**Keywords:** tobacco control, public health, health policy, secondhand smoke, smoking ban, taxation

## Abstract

**INTRODUCTION:**

This study assessed public support for new tobacco control measures in Poland, including a smoking ban on private balconies, regular tobacco tax increases, and a total ban on tobacco sales.

**METHODS:**

A nationwide cross-sectional survey was conducted in 2024 using a computer-assisted web interview (CAWI). The 1080 adults (aged 18–82 years) were interviewed, of which 53% were females. A self-prepared questionnaire included questions on support for various tobacco control measures. The primary outcomes were levels of support for each proposed measure. Attitudes were measured using a 5-point Likert scale, and independent variables included sociodemographic factors and smoking status.

**RESULTS:**

The smoking ban on private balconies was supported by 44.1% of respondents, with higher support among older adults, non-smokers, and those with higher education. Support for annual tobacco tax increases was 47.1%, particularly among younger and middle-aged adults, the educated, and non-smokers. A total of 41.8% of respondents declared support for the total ban on tobacco sales. Higher education, non-smoking status, and voluntary smoke-free home rules were significantly associated (p<0.05) with higher support for all three tobacco control measures. There was no significant impact (p>0.05) of the gender, financial status, household size, and location of the place of residence on public support of analyzed tobacco control measures.

**CONCLUSIONS:**

This study revealed that less than half of adults in Poland declare support for extensive tobacco regulations such as a smoking ban on private balconies, taxation increases, and a ban on tobacco sales. Educational level and smoking status are significantly associated with public attitudes toward tobacco control measures.

## INTRODUCTION

Smoking is widely recognized as a significant risk factor for numerous diseases, contributing substantially to global morbidity and mortality rates^1^. Epidemiological studies have consistently demonstrated a strong association between smoking and different health conditions, including cardiovascular diseases, respiratory conditions, and cancers^2-5^. The health burden of smoking extends beyond active smokers to those exposed to secondhand smoke, also known as passive smoking^5,6^. Secondhand smoke contains many of the same harmful chemicals inhaled by smokers, including nicotine, tar, and carbon monoxide, which pose significant health risks to non-smokers^6^. Children are particularly vulnerable to the effects of secondhand smoke^7^.

The pervasive and involuntary nature of secondhand smoke exposure underscores the importance of stringent smoke-free policies and public health interventions to protect non-smokers from these substantial health hazards. The cumulative evidence underscores the extensive health burden of smoking and passive smoking, including the use of novel tobacco products such as e-cigarettes and heated tobacco^8^.

To mitigate the adverse health impacts associated with tobacco use, a variety of tobacco control measures have been instituted globally. These measures are encapsulated in the MPOWER framework developed by the World Health Organization (WHO)^9^, which encompasses strategies such as monitoring tobacco use, protecting people from tobacco smoke, offering help to quit, warning about the dangers of tobacco, enforcing bans on tobacco advertising, promotion, and sponsorship, and raising taxes on tobacco products^10^. Among these strategies, legal interventions have been particularly effective in curbing tobacco use^11^. These legal changes are designed not only to reduce the accessibility and consumption of nicotine products but also to protect non-smokers from the detrimental effects of passive smoking.

Poland is a prominent example within Central and Eastern Europe for its proactive approach to tobacco control^12^. In 2010, Poland implemented a comprehensive ban on smoking in public places^13^, signifying a critical development in its public health policy. This legislative action was augmented in 2016 with the regulation of e-cigarettes, including a prohibition on their use in public spaces^14^. These measures positioned Poland at the forefront of tobacco control in the region. However, since these initial actions, no substantial legal reforms have been undertaken, except those required to comply with European Union directives, such as the ban on menthol cigarettes and regulations ensuring the quality of tobacco products^12^.

There is a high smoking prevalence in Poland, reaching one-quarter of the adult population^15^. Moreover, 5.9% of adults are daily e-cigarette users, and 4.9% use heated tobacco daily^15^. Poland has an ongoing public debate regarding the potential amendment of its anti-tobacco laws. The first smoking ban in public places was implemented in 1996 (in healthcare facilities, educational entities, and workplaces), significantly extending to numerous public places in 2010. In 2016, e-cigarettes and heated tobacco products were regulated and covered by a ban on their use in public places^12,16^. A focal point of this debate is the consideration of further tightening restrictions on smoking in public places and further increase of taxation of tobacco products. Public health experts raise concerns about the lack of a comprehensive public health strategy for tobacco control in Poland^12^. Moreover, compliance with the ban on smoking in public places, especially with a ban on e-cigarette use, poses a challenge^16^. Findings from the European Tobacco Control Scale 2021 showed that despite significant progress in tobacco control in 1996, Poland is currently lagging behind other Eastern European countries such as Slovenia, Lithuania, and Czechia^17^.

This study investigated public attitudes towards two foreseeable measures (a smoking ban on balconies and increased taxation) and an end-game scenario (a total ban on tobacco) in Poland. Therefore, it provides policymakers with evidence-based insights into public sentiment and preferences regarding smoking restrictions.

## METHODS

### Study design and sample

A nationwide cross-sectional survey was conducted during 2–4 February 2024, utilizing the computer-assisted web interviewing (CAWI) method. The survey comprised 14 closed questions regarding the usage of various nicotine-containing products. The specialized public opinion research company managed data collection, Nationwide Research Panel Ariadna, based in Warsaw, Poland, on behalf of the authors who provided the scientific framework for the study.

A non-probability quota sampling technique was utilized, incorporating a stratification model that considered gender, age, location of residence, and population size. This stratification was established using sociodemographic data from the Central Statistical Office of the Republic of Poland in Warsaw and was representative of the adult population of Poland.

To provide consistency with previous research, cigarette smoking, e-cigarette use, or heated tobacco use was based on the history of past 30-day use of these products (even once). Voluntary smoke-free home rules (total ban) were based on the question: ‘Is tobacco smoked in your home?’ (yes, without limitations; yes, in some parts; no, my home is 100% smoke-free). Sociodemographic questions and questions on smoking were based on previously published data within the same research project, “Poles’ attitudes towards smoking”^15^.

The Ethical Review Board approved this study at the Centre of Postgraduate Medical Education, decision number 403/2023 as of 23 August 2023. All procedures were in line with the Declaration of Helsinki.

### Public attitudes toward selected tobacco control measures

Respondents were asked about their attitudes towards selected tobacco control measures using the following questions: 1) ‘Do you support the introduction of a smoking ban (tobacco use) on the balconies in a multi-family buildings (e.g. in an apartment block or multi-dwelling unit)?’; 2) ‘Do you support the introduction of a regular increase in the tax on tobacco products (e.g. a 10% tax increase every year)?; and 3) ‘Would you support a total ban on the production and sale of cigarettes and other tobacco products?’.

Participants were asked to declare their attitudes towards the abovementioned tobacco control measures on a 5-point Likert scale: ‘definitely yes’, ‘rather yes’, ‘rather no’, ‘definitely no’, and ‘I do not know’. For regression analyses, responses ‘definitely yes’ and ‘rather yes’ were combined into the category: participants who declare support for implementing a particular ban.

### Data analysis

The data were analyzed using IBM SPSS Statistics version 29. Descriptive statistics were employed to present the distribution of categorical variables. Cross-tabulation with a chi-squared test was conducted to compare categorical variables. Regression analyses were used to examine the associations between sociodemographic characteristics and public support for: 1) a smoking ban on private balconies; 2) the introduction of a regular increase in the tax on tobacco products; and 3) a total ban on the production and sale of cigarettes and other tobacco products. Sociodemographic factors and smoking status were considered independent variables. Variables statistically significant in univariable logistic regression were included in the multivariable logistic regression. The strength of association was determined by the odds ratio (OR) and 95% confidence intervals (CI). Statistical significance was defined as p<0.05.

## RESULTS

### Sociodemographic characteristics of the study population

Data were collected from 1080 adults, with mean ± SD age of 48.4 ± 15.5 years. The gender distribution showed a slight majority of females at 53.0%, with the largest age group those aged ≥60 years (30.1%) followed by 30–39 years (19.5%), and 43.4% of the respondents held a university degree. The demographic characteristics of the study population are detailed in Table 1.

**Table 1:** Characteristics of the study population, cross-sectional survey, Poland, February 2024 (N=1080)

*Characteristics*	*n (%)*
**Gender**	
Women	572 (53.0)
Men	508 (47.0)
**Age** (years)	
18–29	140 (13.0)
30–39	211 (19.5)
40–49	201 (18.6)
50–59	203 (18.8)
≥60	325 (30.1)
**University degree**	
Yes	469 (43.4)
No	611 (56.6)
**Occupational status**	
Currently employed or self-employed	656 (60.7)
Pensioner/student/unemployed	424 (39.3)
**Self-declared financial status**	
High	330 (30.6)
Medium	606 (56.1)
Low	144 (13.3)
**Living alone**	
Yes	134 (12.4)
No	946 (87.6)
**Children in home**	
Yes	345 (31.9)
No	735 (68.1)
**Residence**	
Rural	416 (38.5)
City <20000 inhabitants	137 (12.7)
City 20000–99999	211 (19.5)
City 100000–499999	187 (17.3)
City >500000	129 (11.9)
**Cigarette smoking**	
Yes	328 (30.4)
No	752 (69.6)
**E-cigarette or heated tobacco use**	
Yes	198 (18.3)
No	882 (81.7)
**Voluntary smoke-free home rules** (total ban)	
Yes	665 (61.6)
No	415 (38.4)

### Attitudes toward various tobacco control measures

Support for introducing a smoking ban on private apartment balconies was 44.1% (26.0% definitely in favor, 18.1% rather in favor). Conversely, 19.0% were rather against, 21.9% definitely against, and 15.1% were undecided. Support for a regular annual tax increase on tobacco products was shown by 47.1% (28.4% definitely in favor, 18.7% rather in favor), while 15.1% were rather against, 21.4% definitely against, and 16.4% undecided. The least supported option was a total ban on the production and sale of cigarettes and other tobacco products, with 41.8% in favor (25.6% definitely yes, 16.2% rather yes), the strongest opposition with19% of the respondents rather against and 23.3% definitely against, and 15.8% undecided.

General support for studied tobacco control measures is presented in Table 2. Public attitudes towards selected tobacco control measures revealed significant sociodemographic differences (p<0.05), mostly by age, education level, smoking status, e-cigarette or heated tobacco use, as well as voluntary smoke-free policies at home (Table 3).

**Table 2:** Public attitudes towards selected tobacco control measures, cross-sectional survey, Poland, February 2024 (N=1080)

*Support for selected tobacco control measures*	*n (%)*
**Do you support the introduction of a smoking ban on the balcony of a private apartment (e.g. in an apartment block or multi-family building)?**	
Definitely yes	281 (26.0)
Rather yes	195 (18.1)
Rather no	205 (19.0)
Definitely no	236 (21.9)
I do not know	163 (15.1)
**Do you support the introduction of a regular increase in the tax on tobacco products (e.g. a 10% tax increase every year)?**	
Definitely yes	307 (28.4)
Rather yes	202 (18.7)
Rather no	163 (15.1)
Definitely no	231 (21.4)
I do not know	177 (16.4)
**Would you support a total ban on the production and sale of cigarettes and other tobacco products?**	
Definitely yes	277 (25.6)
Rather yes	175 (16.2)
Rather no	205 (19.0)
Definitely no	252 (23.3)
I do not know	171 (15.8)

**Table 3:** Sociodemographic differences in public attitudes towards selected tobacco control measures, crosssectional survey, Poland, February 2024 (N=1080)

*Variables*	*Support for the smoking ban on the private balconies (definitely yes or rather yes)*		*Support for the introduction of a regular increase in the tax on tobacco products (definitely yes or rather yes)*		*Support for the total ban on the production and sale of cigarettes and other tobacco products (definitely yes or rather yes)*	
	*n (%)*	*p*	*n (%)*	*p*	*n (%)*	*p*
**Gender**		0.4		0.4		0.4
Women	259 (45.3)		277 (48.4)		246 (43.0)	
Men	217 (42.7)		232 (45.7)		206 (40.6)	
**Age** (years)		**0.01**		**0.02**		**0.04**
18–29	49 (35.0)		66 (47.1)		51 (36.4)	
30–39	91 (43.1)		118 (55.9)		105 (49.8)	
40–49	87 (43.3)		99 (49.3)		88 (43.8)	
50–59	82 (40.4)		81 (39.9)		74 (36.5)	
≥60	167 (51.4)		145 (44.6)		134 (41.2)	
**University degree**		**0.001**		**<0.001**		**<0.001**
Yes	233 (49.7)		267 (56.9)		229 (48.8)	
No	243 (39.8)		242 (39.6)		223 (36.5)	
**Occupational status**		0.3		**0.004**		0.4
Currently employed or self-employed	281 (42.8)		332 (50.6)		281 (42.8)	
pensioner/student/unemployed	194 (46.0)		177 (41.7)		171 (40.3)	
**Self-declared financial status**		0.5		0.1		0.3
High	153 (46.4)		165 (50.0)		148 (44.8)	
Medium	264 (43.6)		286 (47.2)		250 (41.3)	
Low	59 (41.0)		58 (40.3)		54 (37.5)	
**Living alone**		0.5		0.6		0.3
Yes	55 (41.0)		60 (44.8)		50 (37.3)	
No	421 (44.5)		449 (47.5)		402 (42.5)	
**Children in home**		0.5		0.8		0.7
Yes	147 (42.6)		161 (46.7)		147 (42.6)	
No	329 (44.8)		348 (47.3)		305 (41.5)	
**Residence**		0.08		0.5		0.7
Rural	166 (39.9)		183 (44.0)		164 (39.4)	
City <20000 inhabitants	59 (43.1)		68 (49.6)		55 (40.1)	
City 20000–99999	95 (45.0)		98 (46.4)		92 (43.6)	
City 100000–499999	98 (52.4)		94 (50.3)		84 (44.9)	
City >500000	58 (45.0)		66 (51.2)		57 (44.2)	
**Cigarette smoking**		**<0.001**		**<0.001**		**<0.001**
Yes	86 (26.2)		62 (18.9)		65 (19.8)	
No	390 (51.9)		447 (59.4)		387 (51.5)	
**E-cigarette or heated tobacco use**		**0.01**		**<0.001**		**<0.001**
Yes	70 (35.4)		49 (24.7)		47 (23.7)	
No	406 (46.0)		460 (52.2)		405 (45.9)	
**Voluntary smoke-free home rules (total ban)**		**<0.001**		**<0.001**		**<0.001**
Yes	349 (52.5)		395 (59.4)		331 (49.8)	
No	127 (30.6)		114 (27.5)		121 (29.2)	

### Factors associated with attitudes toward various tobacco control measures

A logistic regression revealed socioeconomic factors influencing public attitudes toward tobacco control measures (Table 4). Age ≥60 years (OR=2.12; 95% CI: 1.37–3.28; p<0.001), having a university degree (OR=1.37; 95% CI: 1.06–1.77; p<0.05), non-smoking status (OR=2.54; 95% CI: 1.80–3.57; p<0.001) and voluntary smoke-free home rules (OR=1.75; 95% CI: 1.29–2.36; p<0.001) were significantly associated with support for the smoking ban on the private balcony. Having a university degree (OR=1.71; 95% CI: 1.30–2.24; p<0.001), current employment or self-employment (OR=1.43; 95% CI: 1.12–1.83; p<0.01), non-smoking status (OR=4.19; 95% CI: 2.93–5.99; p<0.001), and voluntary smoke-free home rules (OR=1.93; 95% CI: 1.41-2.63; p<0.001) were significantly associated with support for the introduction of a regular increase in the tax on tobacco products. Having a university degree (OR=1.51; 95% CI: 1.17–1.96; p<0.01) and non-smoking status (OR=3.31; 95% CI: 2.33–4.71; p<0.001) were significantly associated with support for the total ban on the production and sale of cigarettes and other tobacco products (Table 4). There was no significant impact of gender, financial status, household size and composition, or place of residence, on public support of analysis of tobacco control measures.

**Table 4:** Logistic regression of factors associated with public attitudes towards selected tobacco control measures, cross-sectional survey, Poland, February 2024 (N=1080)

*Variables*	*Support for the smoking ban on the private balcony (definitely yes or rather yes)*		*Support for the introduction of a regular increase in the tax on tobacco products (definitely yes or rather yes)*		*Support for the total ban on the production and sale of cigarettes and other tobacco products (definitely yes or rather yes)*	
	*Univariable*	*Multivariable*	*Univariable*	*Multivariable*	*Univariable*	*Multivariable*
	*OR (95% CI)*	*OR (95% CI)*	*OR (95% CI)*	*OR (95% CI)*	*OR (95% CI)*	*OR (95% CI)*
**Gender**						
Women	1.11 (0.87–1.41)		1.12 (0.88–1.42)		1.11 (0.87–1.41)	
Men ®	1		1		1	
**Age** (years)						
18–29 ®	1	1	1		1	
30–39	1.41 (0.91–2.19)	1.45 (0.92–2.30)	1.42 (0.93–2.19)		0.82 (0.57–1.17)	
40–49	1.42 (0.91–2.21)	1.60 (1.00–2.56)	1.09 (0.71–1.68)		1.11 (0.78–1.58)	
50–59	1.26 (0.81–1.97)	1.50 (0.94–2.39)	0.74 (0.48–1.15)		1.41 (0.99–2.00)	
≥60	1.96 (1.30–2.96)**	2.12 (1.37–3.28)***	0.90 (0.61–1.34)		0.82 (0.54–1.23)	
**University degree**						
Yes	1.50 (1.17–1.91)**	1.37 (1.06–1.77)*	2.02 (1.58–2.57)***	1.71 (1.30–2.24)***	1.66 (1.30–2.12)***	1.51 (1.17–1.96)**
No ®	1	1	1	1	1	1
**Occupational status**						
Currently employed or self-employed	0.88 (0.69–1.13)		1.43 (1.12–1.83)**	1.54 (1.17–2.03)**	1.11 (0.87–1.42)	
Pensioner/student/unemployed ®	1		1	1	1	
**Self-declared financial status**						
High	1.25 (0.84–1.85)		1.48 (0.99–2.21)		1.36 (0.91–2.02)	
Medium	1.11 (0.77–1.61)		1.33 (0.92–1.92)		1.17 (0.81–1.70)	
Low ®	1		1		1	
**Living alone**						
Yes	0.87 (0.60–1.25)		0.90 (0.62–1.29)		0.81 (0.56–1.17)	
No ®	1		1		1	
**Children in home**						
Yes	0.92 (0.71–1.19)		0.97 (0.75–1.26)		1.05 (0.81–1.36)	
No ®	1		1		1	
**Residence**						
Rural	0.81 (0.55–1.21)		0.75 (0.51–1.11)		0.82 (0.55–1.23)	
City <20000 inhabitants	0.93 (0.57–1.50)		0.94 (0.58–1.52)		0.85 (0.52–1.38)	
City 20000–99999	1.00 (0.65–1.56)		0.83 (0.53–1.28)		0.98 (0.63–1.52)	
City 100000–499999	1.35 (0.86–2.11)		0.97 (0.62–1.51)		1.03 (0.66–1.62)	
City >500000 ®	1		1		1	
**Cigarette smoking**						
Yes ®	1	1	1	1	1	1
No	3.03 (2.28–4.03)***	2.54 (1.80–3.57)***	6.29 (0.46–8.59)***	4.19 (2.93–5.99)***	4.29 (3.16–5.83)***	3.31 (2.33–4.71)***
**E-cigarette or heated tobacco use**						
Yes ®	1	1	1	1	1	1
No	1.56 (1.13–2.15)**	0.69 (0.47–1.02)	3.32 (2.34–4.70)***	1.49 (0.99–2.24)	2.73 (1.92–3.88)***	1.45 (0.97–2.15)
**Voluntary smoke-free home rules (total ban)**						
Yes	2.51 (1.93–3.24)***	1.75 (1.29–2.36)***	3.86 (2.96–5.04)***	1.93 (1.41–2.63)***	2.41 (1.86–3.13)***	1.27 (0.94–1.73)
No ®	1	1	1	1	1	1

Variables statistically significant in the univariable logistic regression were included in the multivariable logistic regression. ® Reference categories.

* p<0.05.

** p<0.01.

*** p<0.001.

## DISCUSSION

This study provides critical insights into public support for various tobacco control measures in Poland, focusing on three policy options: a smoking ban on private balconies, a regular increase in tobacco taxation, and a total ban on tobacco sales. The findings in this study demonstrate varied public support for these measures, with nearly half of the respondents supporting each policy. A smoking ban on balconies of private apartments in multi-family buildings was supported by 44.1% of respondents, while 40.9% opposed it, and 15.1% were undecided. A regular annual 10% tax increase on tobacco products garnered 47.1% support, with 36.5% opposed and 16.4% uncertain. The least supported measure, a total ban on the production and sale of tobacco products, was favored by 41.8%, with the highest (42.3%) opposition and 15.8% undecided.

The smoking ban on private balconies is a targeted restriction to reduce secondhand smoke exposure in shared living environments. Such exposure is a risk factor, especially for minors^18^. It is also believed to be a driving factor for increased medical costs^19^. The complicated legal status of such spaces (private, semi-private) and the history of using them as designated go-to places for smokers, complicate the introduction of such measures^20^. In the current study, this measure gathered the support of 44.1% of respondents, particularly among non-smokers, older adults, and those with voluntary smoke-free home rules. The observed support level corresponds with other studies analyzed in a literature review by Boderie et al.^21^ who found an average support level of 41% for a smoking ban in outdoor private areas (e.g. private balconies). Higher acceptance rates among non-smokers and those with voluntary smoke-free home rules are consistent with the results of other studies that showed current smokers to be most skeptical towards any new tobacco control measures^22^.

The second policy option, a regular increase in tobacco taxation, is a widely recognized strategy for reducing smoking prevalence by making tobacco products less affordable^23^. Currently, the WHO recommends a minimum 75% tax share of the retail price of tobacco. This aligns with existing literature, which consistently demonstrates that tax increases are among the most effective tobacco control strategies, particularly in reducing smoking rates among younger and lower income populations^24,25^. In this study, 47.1% of respondents supported this measure. Other studies show higher support for increased taxation of tobacco products, as high as 59% in Denmark^26^ and over 76% in Vietnam^27^. However, the factors associated with this support are not consistent. In the current study, higher acceptance was associated with non-smoking, higher education, and being active in the labor market. Higher education and a non-smoking status are most often associated with higher support for tax increases^26,27^. At the same time, some studies show its dependence on gender^26^, place of residence^27^, or financial status^28^, which did not manifest in the current study. In this study, active occupational status was significantly associated with support for tobacco taxation, and this association was not observed when related to the ban on smoking on balconies or the total ban on sales. This observation requires further investigation.

The third policy option, a total ban on the sale of tobacco products, received the lowest support at 41.8%, with significant opposition. This reflects the controversial nature of such a measure, which, while potentially effective in eradicating smoking, raises concerns about feasibility and public acceptance^29^. Other studies have similarly found that while there is a growing interest in such bans, particularly in contexts of high smoking-related mortality, public support for this so-called endgame scenario is often lower than for other policy options. A recent study on support for a tobacco endgame strategy in 18 European countries^30^ revealed that acceptance levels vary between 20% and 60%, with a significant inverse trend observed with age and education level. Regarding the influence of education level, those results are contrary to the findings of the current study, where having a university degree was found to be significantly correlated with support for a total ban on the sale of tobacco products.

Findings from the EUREST-PLUS study showed that among smokers and recent quitters in Europe, 50.5% declared high support for implementing measures further to regulate tobacco products, and the majority of smokers would support a ban on tobacco products in the future if the government provided assistance to quit smoking^31^. Moreover, among smokers in Europe, public support for a ban on e-cigarette use in public places increased from 53.1% in 2016 to 54.6% in 2018, with the highest increase in Greece^32^. Data from Hungary showed that regulatory tightening of the Hungarian tobacco retail market resulted in short-term reductions in youth smoking prevalence^33^. Experiences from the European countries underline the need for a unified European tobacco control strategy implemented at the European Union level.

The practical implications of these findings are significant for policymakers. The relatively strong support for a smoking ban on private balconies suggests a potential area for immediate policy action, particularly in urban settings where shared living spaces are common. Additionally, the support for increased tobacco taxation suggests that this could be a politically viable and effective measure to further reduce smoking rates in Poland.

However, the mixed support for a total tobacco ban highlights the need for a more nuanced approach, possibly involving phased implementation or complementary strategies to address public concerns.

### Limitations

The study is subject to several limitations. Firstly, the CAWI sampling strategy only provided representative data for the demographic structure of the adult population, excluding adolescents from the study population. Secondly, there is a potential for recall and demand biases, as all data were self-reported. Third, the non-probabilistic sampling is also a limitation of this study.

## CONCLUSIONS

This study revealed that less than half of adults in Poland declare support for extensive tobacco regulations such as a smoking ban on private balconies, taxation increases, and a ban on tobacco sales. Social support for all analyzed policies was influenced primarily by education level, smoking status, and voluntary smoke-free rules at home. The significant association between voluntary smoke-free home rules and support for stricter policies indicates that individuals who already practice self-imposed smoking restrictions are more likely to endorse broader regulatory measures. Further educational activities are needed to build social support for tobacco control measures in Poland.

## Data Availability

The data supporting this research are available from the authors on reasonable request.
